# Effect of the TLR9 signaling pathway on acyclovir infection with herpes simplex virus type 2 in HaCaT cells

**DOI:** 10.3389/fmicb.2025.1560340

**Published:** 2025-03-24

**Authors:** Jialing Shi, Lin Kuang, Li Qi, Ruoyu Li, Yangfan Wu

**Affiliations:** ^1^School of Integrated Chinese and Western Medicine, Hunan University of Chinese Medicine, Changsha, China; ^2^Xiangxing College of Hunan University of Chinese Medicine, Changsha, China

**Keywords:** HSV-2, acyclovir, TLR9, RNA-seq, HaCaT

## Abstract

**Introduction:**

The objective of this study was to investigate the effect of acyclovir (ACV) on the TLR9 signaling pathway after human immortalized epidermal (HaCaT) cell infection with herpes simplex virus type 2 (HSV-2).

**Methods:**

In this study, an *in vitro* cell model of HSV-2 infection was successfully constructed by infecting HaCaT with HSV-2 virus. In order to explore the antiviral mechanism of acyclovir (ACV), high-throughput transcriptome sequencing (RNA-seq) was used to analyze the genome-wide expression profiling of infected cells before and after ACV treatment, and to systematically compare the change characteristics of differentially expressed genes (DEGs). Based on the sequencing results, the study further focused on Toll-like receptor (TLR) 9 signaling, using quantitative real-time reverse transcriptase chain reaction (qRT-PCR) to quantitatively detect the effect of ACV intervention on the mRNA expression level of key molecules of TLR 9 signaling pathway in HSV-2 infected HaCaT cells.

**Results:**

A total of 896 significant changes in gene expression were identified by the transcriptome analysis, including 314 upregulated genes and 582 downregulated genes. GO enrichment analysis showed that the differentially expressed genes were mainly related to CC includes the ubiquitin ligase complex, mitochondrial protein-containing complex, DNA-binding transcription activator activity, exonuclease activity, catabolic process, nuclear-transcribed mRNA catabolic process nuclear-transcribed mRNA catabolic process; KEGG enrichment analysis showed that the differentially expressed genes were mainly related to Toll-like receptor signaling pathway, herpes simplex virus 1 infection, and TNF signaling pathway. The RT-PCR results were confirmed to be basically consistent with the sequencing results.

**Conclusion:**

ACV altered the transcriptome level of HSV-2 infection in HaCaT cells. The RT-PCR results confirmed that ACV intervened in HSV-2 infection through the TLR9 signaling pathway.

## 1 Introduction

Genital herpes (GH) is a sexually transmitted disease caused by herpes simplex virus 2 (HSV-2) infection, clinically manifested as erythema, papules, vesicles, erosion and exudation, associated with burning skin and mucous membranes with genital and anal (Parra-Sánchez, 2019). HSV infection is characterized by lifelong infection, recurrent recurrence, and neurotropic (Gatherer et al., 2021). The pathogenesis of genital herpes involves complex virus-host interactions.

Acyclovir (ACV) as a first-line treatment for genital herpes. Belongs to nucleoside analogues that exert antiviral effects through selective inhibition of HSV DNA polymerase (Bourne et al., 2019). It is phosphorylated in infected cells by the viral thymidine kinase in infected cells and converted to an active form, which competitively inhibits viral DNA synthesis (Lee and Ashkar, 2012). Recent studies suggest that acyclovir may enhance the antiviral effect by modulating the host innate immune response. The mechanism of action involves the regulation of the TLR 9 signaling pathway.

Recently, Toll-like receptor 9 (TLR 9) was found to play a key role in the immune response against HSV (Lv et al., 2018). Toll-like receptors (TLRs) are the pattern recognition receptor (Gill et al., 2008) for microbial pathogens. TLR 9 is a key pattern recognition receptor for recognizing viral DNA and is an important component of innate immunity (Feng et al., 2021), which is mainly expressed in epithelial cells and immune cells. After HSV-2 infection, TLR9 recognizes the DNA of viral HSV-2, initiates the innate immune response, activates NF-*κ*B and IRF7 (Gill et al., 2005) through MyD 88-dependent signaling pathway (Gill et al., 2006), and induce production of type I interferon and pro-inflammatory cytokines (Tognarelli et al., 2019).

Transcriptome sequencing (RNA-seq) is a high-throughput sequencing technology that was developed in recent years, which can effectively evaluate the interactions between viruses and host cells (Kumar, 2025). Transcriptome sequencing has been used to analyze gene transcription levels and related mechanisms in different cells and in animal models of HSV infection (Rumpf and Schulz, 2025). In this study, high-throughput RNA sequencing technology was used to perform transcriptomic analysis of ACV-treated HaCaT cells infected with HSV-2, and the differentially expressed genes after ACV-infected HSV-2 infection were screened using virus-infected cells as controls. The functions of differentially expressed genes and the cellular processes and signaling pathways that may be involved in HSV-2 infection were further analyzed. In this study, we explored the mechanism of action of ACV against HSV-2 based on the TLR9 signaling pathway. And the replication efficiency of the virus was observed by determining HSV-2 *gD* levels, an advanced gene product of the virus.

## 2 Materials and methods

### 2.1 Reagents and instruments

#### 2.1.1 Cells and viruses

The HaCaT (Procell CL-0090) cell line was purchased from Procell Life Science & Technology Co., Ltd. HSV-2 was purchased from the Hunan Prema Pharmaceutical Research Center as *human herpesvirus 2/ATCC VR-1779*. Both overexpression and knockdown viruses were constructed by Shanghai Genechem Co., Ltd.

#### 2.1.2 Main reagents

Fetal bovine serum (FBS), MEM medium, penictomycin, and pancreatic enzymes were purchased from Wuhan Pricella Company. An RNA extraction kit was purchased from SIMGEN, and the cDNA reverse transcription and amplification kit was purchased from Novoprotein. Puromycin was purchased from Shanghai Jikai Gene Medical Technology Co., Ltd. Primers were purchased from Beijing Qingke Biotechnology Co., Ltd. Acyclovir tablets were produced by Sichuan Kelun Pharmaceutical Co., Ltd. (Approval Number: Guoyao Zhunzi H10983103).

#### 2.1.3 Main instruments

Secondary biosafety cabinet (Thermo Fisher Scientific, Germany), high-speed refrigerated centrifuge (Thermo Fisher, Germany), carbon dioxide incubator (Thermo, Germany); inverted fluorescence microscope (CarelZeiss, Germany); real-time quantitative PCR instrument (Bio-Rad/GFX Connect); gene amplifier (Eppendorf/Mastercycler nexus GX2, Germany); vertical automatic pressure steam sterilizer (Xiamen Zhi Micro Instrument Company).

### 2.2 Preparation of serum containing drugs

Twenty SPF-grade SD rats were purchased from Hunan SJA Laboratory Animal Co., Ltd. [License Number: SCKX (Xiang) 2019-0004]. There were 10 male and 10 female rats, 8 weeks old, weighing 200 ± 20 g. The rats were maintained in an isolated environment at a temperature of 22–26°C and a humidity of 40–60% and ate and drank freely. After 3 days of adaptive feeding, the animals were divided into a blank control group and an acyclovir group according to the random number table method, with 10 animals in each group. The rat administration dose was calculated based on the equivalent dose ratio of the human and rat body surface areas. Acyclovir was administered at 0.37 mg/kg 3 times each day for 7 days, and the blank control group was given the same volume of normal saline for 2 times each day for 7 days. After fasting for 24 h after the last dose, pentobarbital sodium was intraperitoneally injected. After the rats were completely anesthetized, abdominal aortic blood was collected, and after standing at room temperature for 2 h, the upper serum was collected by centrifugation for 15 min at 3,000 r/min and frozen in a −80°C freezer. The animal experiment operation was approved by the Experimental Animal Ethics Committee of Hunan University of Traditional Chinese Medicine (Approval Number: HNUCM21-2405-19).

### 2.3 Construction and identification of overexpression and knockdown expression

Cells in logarithmic growth and displaying good growth and an appropriate multiplicity of infection were selected according to a pre-experiment, and an appropriate amount of lentiviral vector was added. The negative control group (CON335) of the lentivirus overexpression group (LV-TLR9-L) was transfected with CON335 virus. The negative control group (CON313) of the lentiviral knockdown group (LV-TLR9-H) was transfected with CON313 virus.

After 24 h of infection, the effect of the infection was preliminarily observed by fluorescence microscopy. After 72 h of infection, an appropriate concentration of puromycin and complete medium was added. The solution was changed regularly, and cells were screened for stable transfection. The cells were collected for RT-PCR analysis after 7 days of culture to identify the expression level of the target gene *TLR9*, and the cells with qualified identification results were cryopreserved and passaged for follow-up experiments.

### 2.4 Cell experiments

HaCaT cells were cultured in 10% FBS with 1% penicillin/streptomycin and 89% MEM medium at 37°C and 5% CO2. After 1.25% trypsinization, cells were seeded at a density of 4 × 10^5^/mL in 6-well plates to continue culturing.

Logarithmic cells in the growth phase were seeded in 6-well plates, and when the cell density reached 90%, the following treatments were applied: KBZ: only 2,000 μL complete medium; BLZ: add 50 μL HSV-2 virus solution and 1,950 μL of complete medium; ACV: add 50 μL HSV-2 virus solution, 100 μL ACV of drug-containing serum and 1,850 μL of complete medium; LV-TLR 9-H + H: Hacat cells overexpressing TLR 9 with 50 μL HSV-2 viral solution and 1,950 μL of complete medium; LV-TLR 9-H + HA: Hacat cells overexpressing TLR 9 with 50 μL HSV-2 viral solution, 100 μL ACV of drug-containing serum and 1,850 μL of complete medium; LV-TLR 9-L + H: Hacat cells expressing TLR 9 were supplemented with 50 μL HSV-2 viral solution and 1,950 μL of complete medium; LV-TLR 9-L + HA: Hacat cells with 50 μL HSV-2 virus solution 100 μL, ACV-containing serum and 1,850 μL of complete medium. After intervention, put into the incubator. This experiment was performed with the virus-related operations in the BSL-2 laboratory. Each experiment was repeated 3 times.

### 2.5 RNA-seq analysis and gene expression change analysis

Three samples were selected from the BLZ group and the ACV group for biological replicates, and the RNA extraction kit was used to extract the total RNA. An Agilent 2100 bioanalyzer was used to determine the RNA integrity and total amount. After the quality inspection was confirmed, the cDNA library was sequenced using the Illumina platform for high-throughput sequencing.

### 2.6 RNA-seq technology and analysis of differentially expressed genes and core genes

DESeq2 software was used to analyze differentially expressed genes (DEGs) in BLZ group and ACV group. DEGs satisfied the criteria *p* < 0.05 and |log2(fold change)| >1. ClusterProfiler was used to perform GO and KEGG enrichment analysis of DEGs.

Transcriptomics was performed by the Shanghai Tanpu Biotechnology Co., Ltd. (Shanghai, China).

### 2.7 RT-PCR

When the cell viability was about 60%, the total RNA was extracted with an RNA extraction kit. The RNA extraction kit and reverse transcription kit were used for mRNA extraction and reverse transcription of cDNA from HaCaT cells, respectively, and reverse transcription polymerase chain reaction (RT-PCR) was carried out with cDNA, SYBR master mix, and PCR primers in a 10-μL system. Relative mRNA expression was calculated by PCR using the $2^{-\mathrm{\Delta \Delta CT}}$ method (see Table 1).

**Table 1:** Primer sequences for RT-PCR.

Gene names	Forward primers (5′ → 3′)	Reverse primers (5′ → 3′)
*GAPDH*	GGAGCGAGATCCCTCCAAAAT	GGCTGTTGTCATACTTCTCATGG
*TLR9*	CTGCCTCCTACCCTGTGAG	GGATGCGGTTGGAGGACAA
*MyD88*	GGCTGCTCTCAACATGCGA	CTGTGTCCGCACGTTCAAGA
*NF-κB*	AACAGAGAGGATTTCGTTTCCG	TTTGACCTGAGGGTAAGACTTCT
*gD*	GACCCAAAGACCGACCCACA	CCAAGGCGACCAGACAAACG

### 2.8 Statistical analysis

SPSS 26.0 and GraphPad Prism 8.0 software were used for statistical analysis. The experimental data were used as continuous data, expressed as $\bar{x\;}\pm s$ , and the independent samples *t*-test was used for comparisons between the two groups. One-way ANOVA was used for comparing differences between multiple groups, and *p* < 0.05 was considered statistically significant.

## 3 Results

### 3.1 Transcriptome results

#### 3.1.1 Principal component analysis of gene expression in different groups

**Figure 1:**
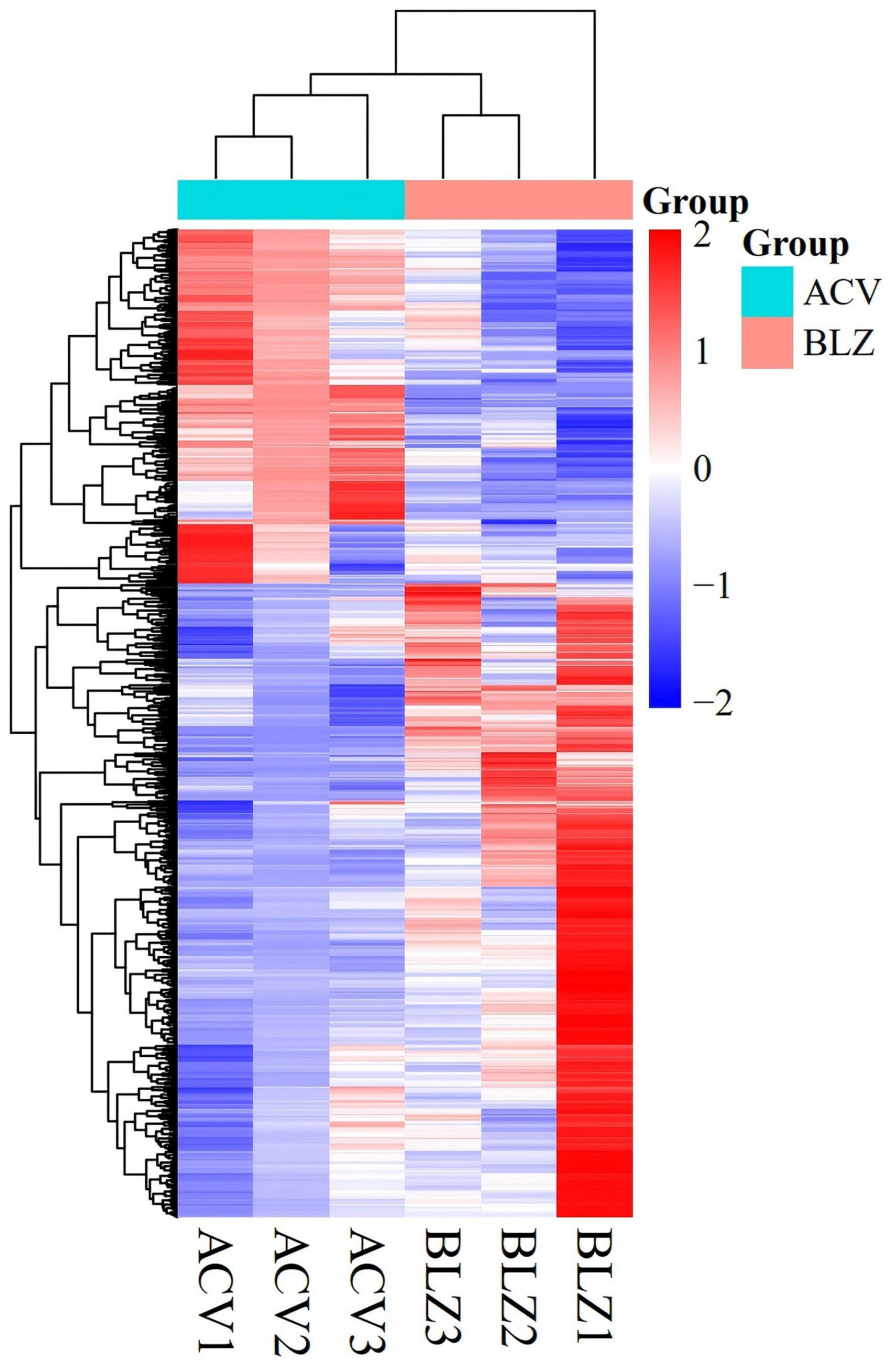
Differential gene cluster plots. According to the standardized expression values of the differentially expressed genes in the samples, the Pearson correlation was calculated, and the systematic clustering method (hierarchical cluster) was used to obtain the overall clustering results of the samples. In the figure, changes in expression are indicated by changes in color, where blue indicates lower expression and red indicates higher expression.

According to the expression value of all genes in each sample: FPKM, the correlation coefficient of samples within and between groups is calculated and drawn into a heat map, which can directly show the sample difference between groups and the sample duplication within groups. The higher the correlation coefficient between samples, the closer the expression pattern is, and the sample correlation heat map is shown in Figure 1.

#### 3.1.2 Differentially expressed genes

**Figure 2:**
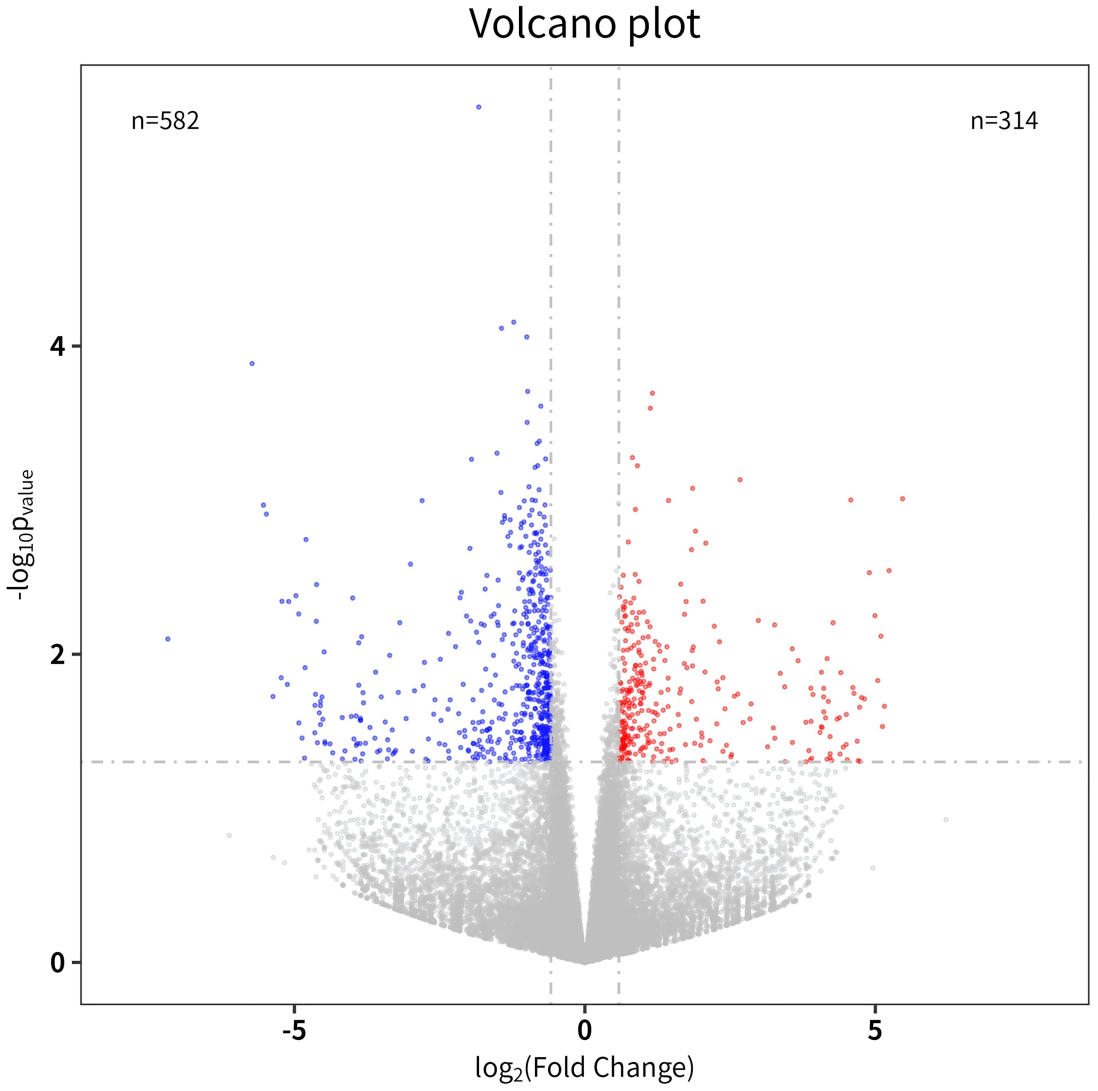
Volcano map. The abscissa is a multiple of the difference (logarithmic transformation with a base of 2), and the ordinate is the *p*-value (logarithmic transformation with a base of 10).

In sequencing of BLZ and ACV genes, DEGs 896 were select 582 downregulated genes and 314 upregulated genes (Figure 2).

#### 3.1.3 GO enrichment analysis

**Figure 3:**
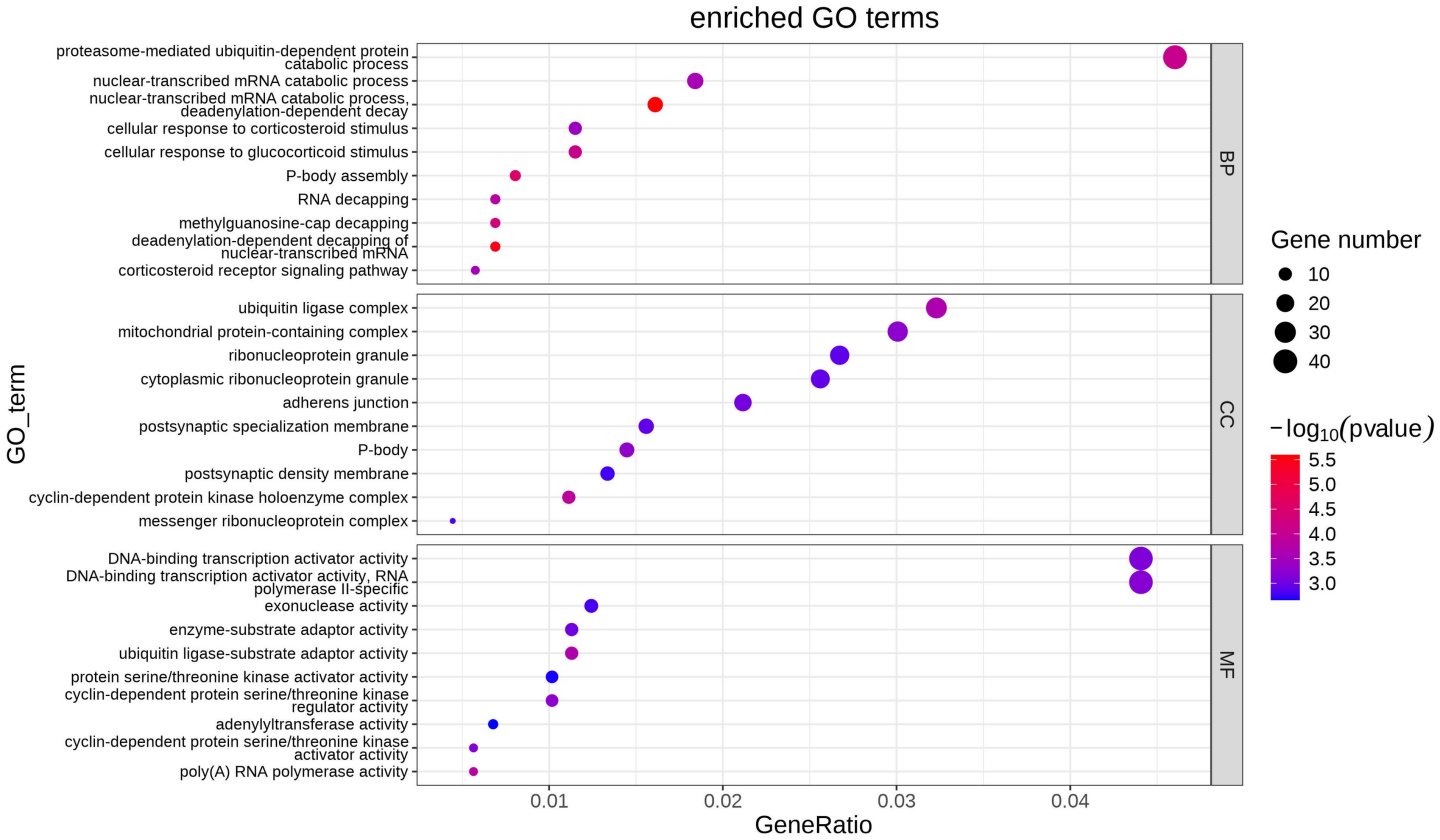
Bubble map of GO term enrichment statistics. Each point indicates the enrichment of the GO entry, and the closer the color is to the red, the higher the enrichment is. The size of each dot indicates the number of genes enriched to that GO entry, and larger dots indicate more genes enriched to that GO entry, and less vice versa.

GO functional enrichment analysis was performed from biological process (BP), cellular component (CC) and molecular function (MF). The main enrichment of DEGs is shown in Figure 3. CC includes the ubiquitin ligase complex, mitochondrial protein-containing complex, cytoplasmic ribonucleoprotein granule, etc. MF includes the DNA-binding transcription activator activity, DNA-binding transcription activator activity, RNA polymerase II-specific, exonuclease activity, ubiquitin ligase-substrate adaptor activity, etc. BP includes proteasome-mediated ubiquitin-dependent protein, catabolic process, nuclear-transcribed mRNA catabolic process nuclear-transcribed mRNA catabolic process, cellular response to glucocorticoid stimulus cellular response to corticosteroid stimulus, etc.

#### 3.1.4 KEGG enrichment analysis

**Figure 4:**
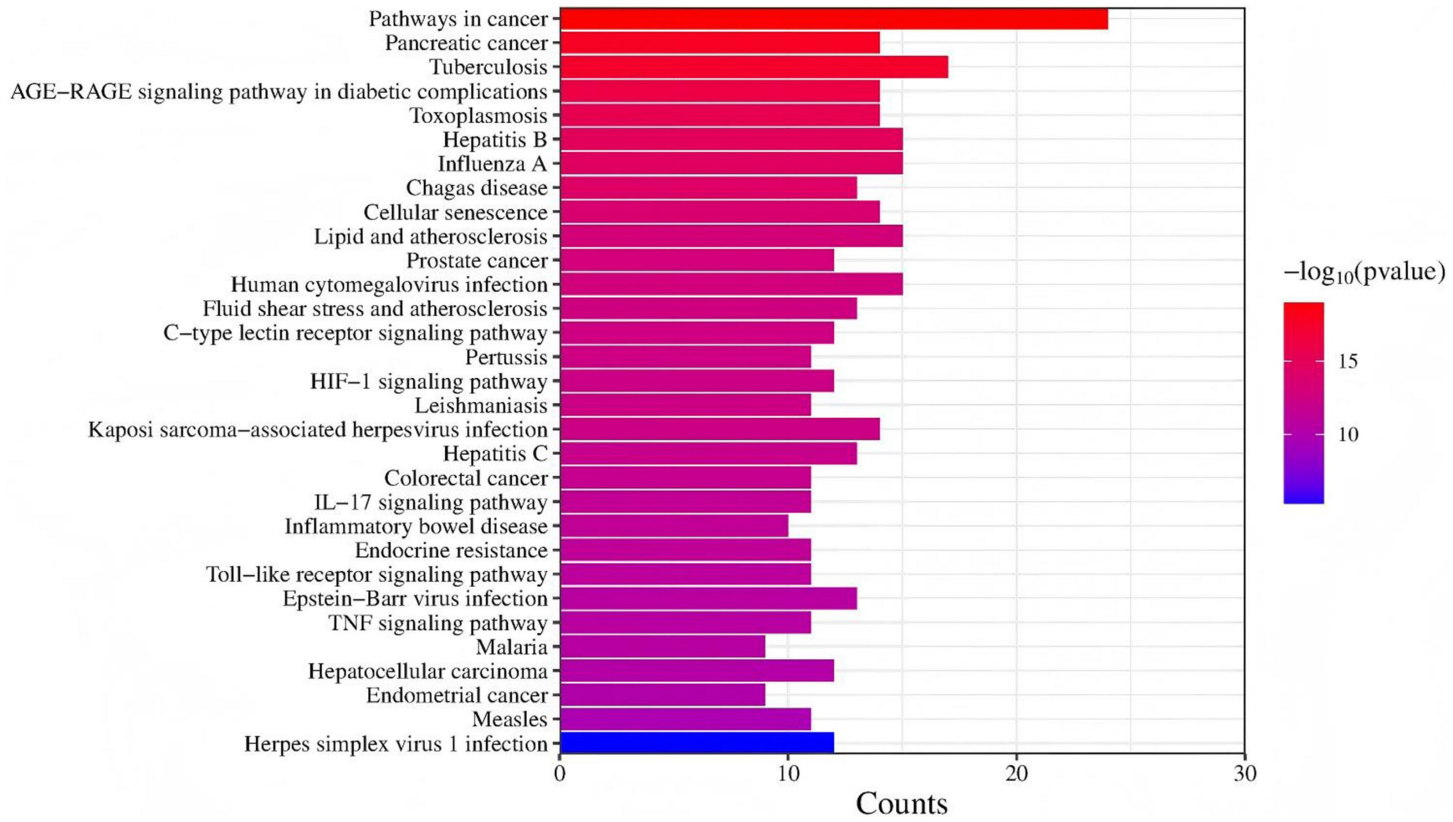
The KEGG pathway annotation results. The KEGG entries enriched in all samples were taken for analysis, the ordinate is KEGG entries, the abscissa is different sample names, and the closer the color is to red, the higher the enrichment is.

The DEGs KEGG enrichment analysis obtained by screening showed that these DEGs were mainly distributed in 31 pathways, as shown in Figure 4, mainly include: Toll-like receptor signaling pathway, herpes simplex virus 1 infection, TNF signaling pathway, AGE-RAGE signaling pathway in diabetic complications, human cytomegalovirus infection, pertussis, HIF-1 signaling pathway, Kaposi sarcoma-associated herpesvirus infection, Epstein–Barr virus infection, IL-17 signaling pathway, malaria, hepatitis C, measles, pathways in cancer, prostate cancer.

### 3.2 Construction and validation of TLR 9 overexpression and knockdown cell lines

**Figure 5:**
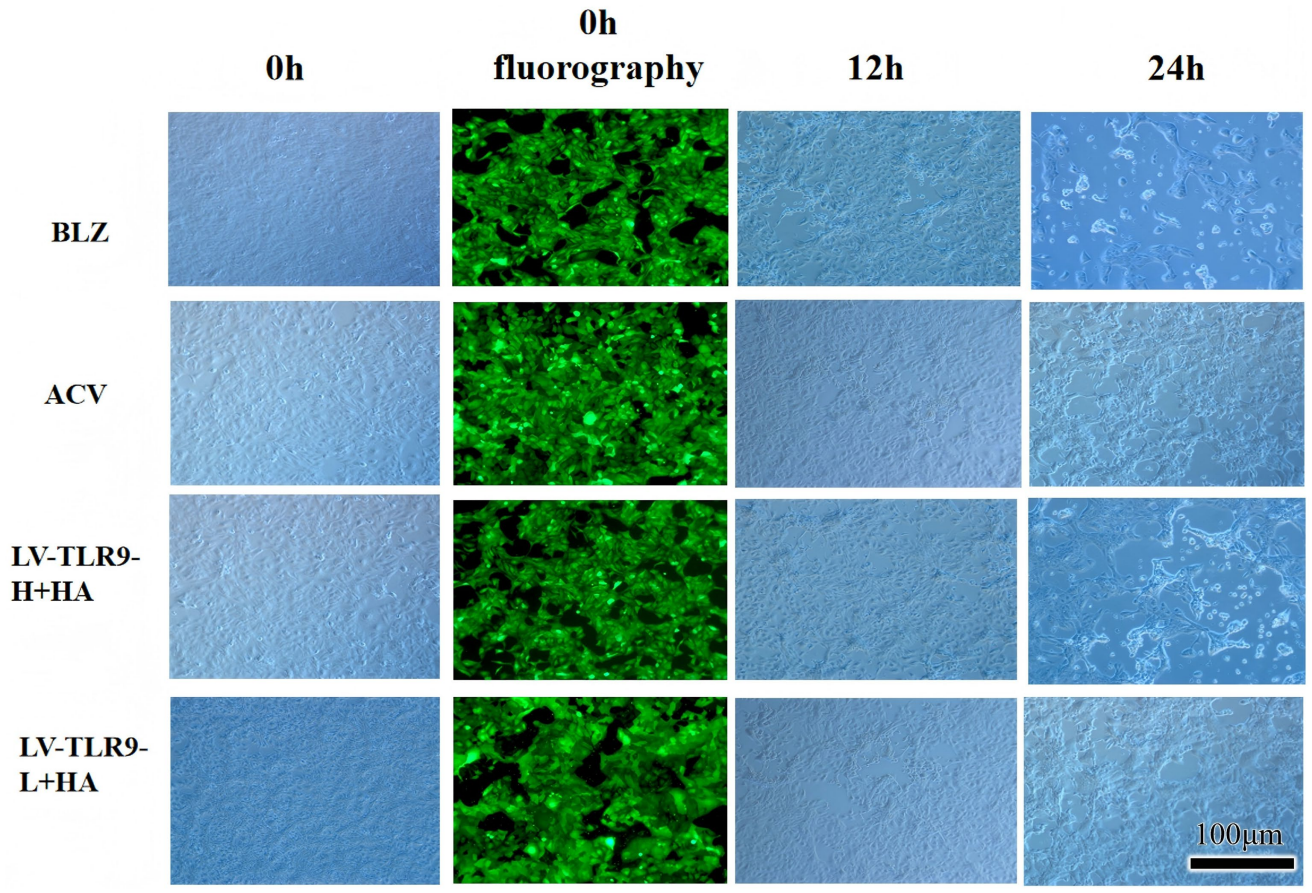
Fluorescence observation of lentiviral transfection.

**Figure 6:**
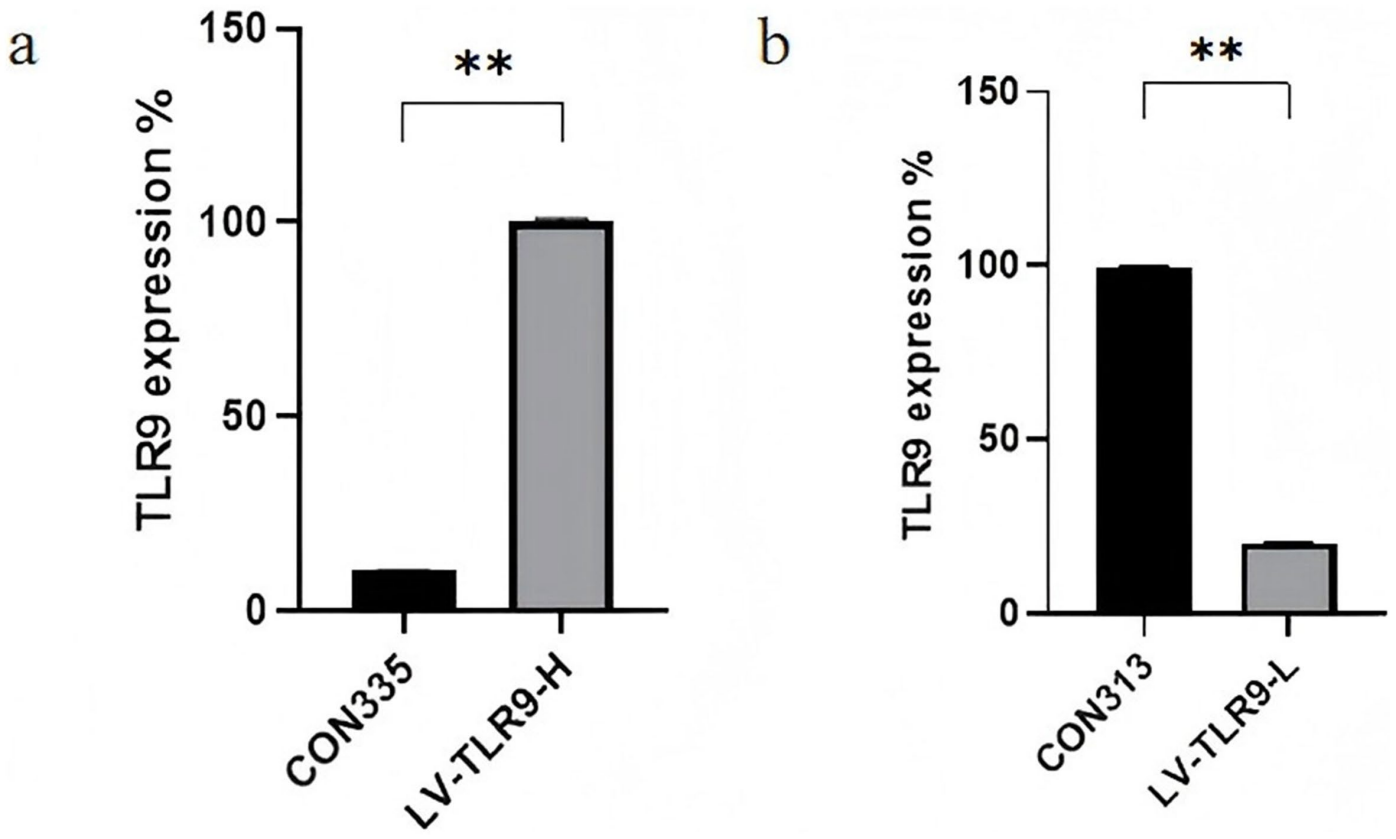
Identification of RNA expression levels in stable transgenic cell lines. **(a)** Construction of TLR 9-overexpressing cells. **(b)** Construction of TLR 9-knockdown cells.

After transfection of HaCaT cells with lentivirus, models were made and cells of each group were observed by fluorescence microscope. The results showed that strong green fluorescence was observed in the transfection groups (Figure 5) and the transfection efficiency was higher. RT-PCR showed that *TLR9* was significantly expressed in the LV-TLR9-H group compared with the CON335 group (Figure 6a). *TLR9* expression was significantly reduced and statistically significantly different in the LV-TLR9-L group compared with the CON313 group (Figure 6b). These results indicated that *TLR9* overexpression and knockdown in HaCaT cell lines had been successfully constructed after lentivirus transfection and screening.

### 3.3 Expression of TLR9 signaling pathway-related genes in HaCaT cells after HSV-2 and ACV intervention

**Figure 7:**
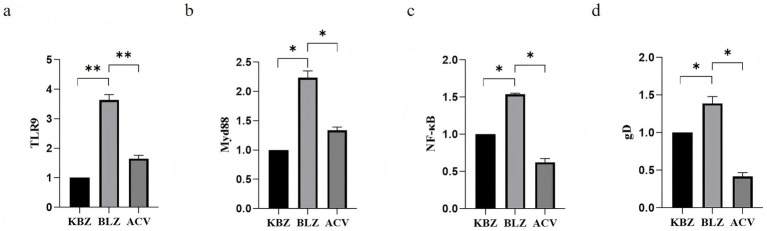
Expression of *TLR9* signaling pathway-related genes after ACV intervention in HSV-2-infected HaCaT cells. **(a)** The expression status of TLR 9. **(b)** The expression status of MyD 88. **(c)** The expression status of NF-κB. **(d)** The expression status of gD.

The difference in expression of the target genes *TLR9*, *MyD88*, *NF-κB*, and *gD* in HSV-2-infected HaCaT cells after ACV intervention was verified by RT-PCR. The results showed that compared with the KBZ group, HSV-2 upregulated *TLR9*, *MyD88*, *NF-κB*, and *gD*, and there were statistically significant differences. Compared with the BLZ group, ACV downregulated the expression of *TLR9*, *MyD88*, *NF-κB*, and *gD* with statistical differences (Figure 7).

### 3.4 Expression of the TLR 9 signaling pathway in overexpressing TLR9 Hacat cells after HSV-2 and ACV intervention

**Figure 8:**
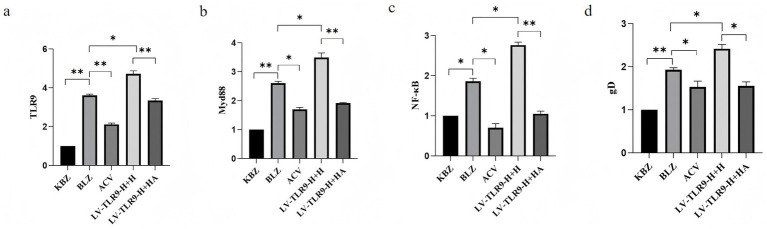
Expression of TLR9 signaling pathway-related genes in HSV-2-infected HaCaT cells overexpressing *TLR9* after ACV intervention. ^*^p < 0.05, ^**^p < 0.01, and ns: *p* > 0.05. **(a)** The expression status of TLR 9. **(b)** The expression status of MyD 88. **(c)** The expression status of NF-κB. **(d)** The expression status of gD.

After ACV intervention, the differential expression of mRNA TLR9, TLR 9, MyD 88, NF-*κ*B, and gD in HSV-2-overexpressing Hacat cells infected with GAPDH as an internal reference was verified by RT-PCR. Results shown in Figure 8, ACV decreased the expression of TLR 9, MyD 88, NF-*κ*B, gD compared to BLZ (*p* < 0.05). LV-TLR 9-H + H caused the up-regulation of MyD 88 and NF-*κ*B compared to BLZ (*p* < 0.05). Compared with LV-TLR9-H + H group, LV-TLR9-H + HA group could down-regulate the expression of TLR 9, MyD 88, NF-*κ*B, and gD.

### 3.5 Expression of the TLR9 signaling pathway in knockdown TLR9 Hacat cells after HSV-2 and ACV intervention

**Figure 9:**
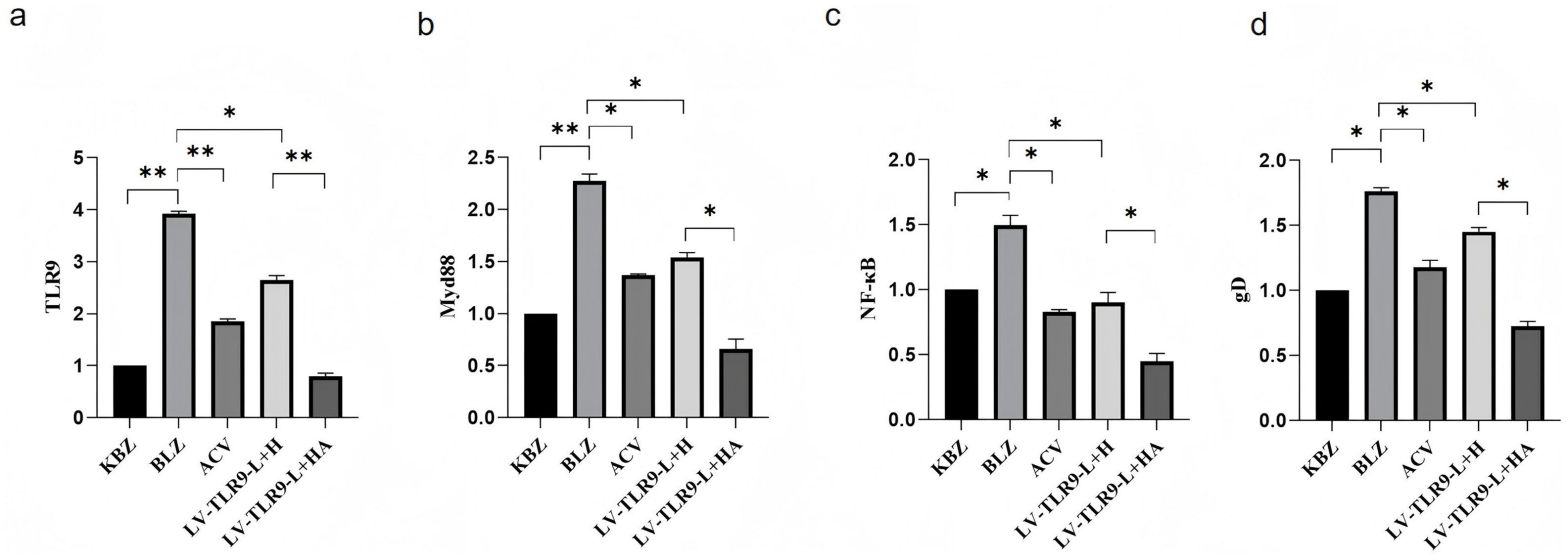
Expression of *TLR9* signaling pathway-related genes in HSV-2-infected HaCaT cells knockdown *TLR9* after ACV intervention. ^*^p < 0.05, ^**^p < 0.01, and ns: *p* > 0.05. **(a)** The expression status of TLR 9. **(b)** The expression status of MyD 88. **(c)** The expression status of NF-κB. **(d)** The expression status of gD.

Using GAPDH as internal reference, RT-PCR verified the differential expression of mRNA TLR9, MyD 88, NF-*κ*B, and gD in Hacat TLR 9 cells infected with HSV-2. As shown in Figure 9, ACV decreased the expression of TLR 9, MyD 88, NF-*κ*B, and gD compared to the BLZ group (*p* < 0.05). Compared with BLZ, LV-TLR 9-L + H, caused the downregulation of TLR 9, MyD 88, NF-κB, and gD (*p* < 0.05). Compared with LV-TLR 9-L + H group, TLR 9, MyD 88, NF-*κ*B, and gD in LV-TLR 9-L + H group (*p* < 0.05).

## 4 Discussion

The body’s immune response to HSV-2 is both innate and acquired (Kurt-Jones et al., 2017). Innate immunity is primarily mediated by leukocytes, such as neutrophils, macrophages, and dendritic cells, which are able to engulf pathogens and coordinate other responses with the synthesis of various inflammatory mediators and cytokines (Lucinda et al., 2017). A wide range of pattern recognition receptor families (PPRs) can help the immune system recognize pathogen-related molecular patterns, such as double-stranded RNA and lipopolysaccharides (Bauer and Wagner, 2002). *TLR9* play an important role in this process.

*TLR9* is involved in the recognition of DNA with unmethylated CpG structures in the genetic material of higher organisms or microorganisms or viruses (Gill et al., 2006). It was originally discovered in bacteria and has characteristic immunomodulatory effects based on the motifs present in bacterial DNA (Takeshita et al., 2004). However, it has since been found to be common in viruses and vertebrates and plays an important role in the recognition of Herpesviridae viruses, such as herpes simplex virus (HSV-1 and HSV-2), cytomegalovirus, and Epstein–Barr virus, and can help trigger a rapid innate immune response (Krug et al., 2004). The *TLR9* receptor requires a non-canonical *NF-κB* pathway for pathogen recognition (Hochrein et al., 2004). Upon ligand binding, the receptor structure changes, and the *MyD88* linker docks to the cytoplasmic domain of *TLR9* and then forms a special complex with kinases of the IRAK family (Latz et al., 2004). This complex triggers a signaling cascade that causes *NF-κB* transcription factors to enter the nucleus, thereby initiating the expression of cytokines and chemokines (van Lint et al., 2010).

TLR9/MyD88/NF-*κ*B signaling pathway is a classical pathway existing in epithelial cells (Zyzak et al., 2020), and its upregulation regulates the secretion of pro-inflammatory factors such as IL-6, TNF-α, and IL-1β *in vivo*, which plays an important role in the pathogenic process of GH (Svensson et al., 2007). MyD88 is involved in signaling in most tlr (Gaajetaan et al., 2012), and downregulation of MyD 88 is able to significantly inhibit HSV-2-mediated activation of AP-1 in reproductive epithelial cells, inhibiting viral replication (Wang et al., 2018). The mechanism of action may be that it suppresses the expression of related proteins on the TLR9/MyD88/NF-κB signaling pathway, and reduces the content of IL-6, TNF-α, and IL-1β inflammatory factors in vivo, thus achieving the effect of reducing skin inflammation (Zhang et al., 2013). The present experiment proved that TLR 9, NF-κB and MyD 88 were significantly lower in epithelial cells of ACV group than that in the model group (Barrow et al., 2024). This indicates that ACV may downregulate the content of proinflammatory factors and reduce skin inflammation by inhibiting the expression of TLR9/MyD88/NF-κB signaling pathway.

We propose that acyclovir not only has direct antiviral effects, but also enhances the host antiviral immune response by regulating the TLR 9 signaling pathway and improves the therapeutic efficacy on genital herpes (Bao and Liu, 2013). Acyclovir may also reduce the disease rate of recurrence by enhancing TLR 9-mediated immune memory. This dual mechanism of action provides a new idea for improving the therapeutic effect of genital herpes (Sauter et al., 2014).

## 5 Conclusion

In summary, we conclude that after HSV-2 infection, *TLR9* effectively stimulates innate immune cells to control HSV-2 infection in HaCaT cells.

## Data Availability

The original data in this study are included in the Supplementary material, further inqueries can be directed to the corresponding author.
